# Personality changes in patients with vestibular dysfunction

**DOI:** 10.3389/fnhum.2013.00678

**Published:** 2013-10-29

**Authors:** Paul F. Smith, Cynthia L. Darlington

**Affiliations:** Department of Pharmacology and Toxicology, School of Medical Sciences, and the Brain Health Research Centre, University of OtagoDunedin, New Zealand

**Keywords:** personality, vestibular disorders, vestibular function tests, derealization, depersonalization, out of body experience

## Abstract

The vestibular system is a sensory system that has evolved to detect linear and angular acceleration of the head in all planes so that the brain is not predominantly reliant on visual information to determine self-motion. Since the vestibular system first evolved in invertebrate species in order to detect gravitational vertical, it is likely that the central nervous system has developed a special dependence upon vestibular input. In addition to the deficits in eye movement and postural reflexes that occur following vestibular dysfunction, there is convincing evidence that vestibular loss also causes cognitive and emotional disorders, some of which may be due to the reflexive deficits and some of which are related to the role that ascending vestibular pathways to the limbic system and neocortex play in the sense of spatial orientation. Beyond this, however, patients with vestibular disorders have been reported to experience other personality changes that suggest that vestibular sensation is implicated in the sense of self. These are depersonalization and derealization symptoms such as feeling “spaced out”, “body feeling strange” and “not feeling in control of self”. We propose in this review that these symptoms suggest that the vestibular system may make a unique contribution to the concept of self through information regarding self-motion and self-location that it transmits, albeit indirectly, to areas of the brain such as the temporo-parietal junction (TPJ).

## Introduction

The balance organs in the inner ear (the “vestibular system”) encode head movement (strictly speaking, head acceleration) and generate rapid eye movement “vestibulo-ocular reflexes (VORs)” and postural reflexes “vestibulo-spinal reflexes (VSRs)” that are important for maintaining clear vision and balance (Cullen, 2012). During unintentional head movement, which can include movement as small as that produced by the pulse beat, the image of the visual world on the retina shifts sufficiently, that without the compensatory eye movements generated by the VORs, the visual world would appear to smear, a condition known as “oscillopsia”. Similarly, the VSRs generate compensatory postural adjustments during head movement in order to maintain balance. Humans with deficient vestibular function experience difficulty maintaining balance and consequently cannot walk properly (“ataxia”; Cullen, 2012). Recent epidemiological studies suggest that vestibular disorders occur in more than 35% of adults aged 40 or older in the USA (Agrawal et al., 2009). Saber Tehrani et al. (2013) have recently estimated that, of 3.9 million patients visiting a Hospital Emergency Department for dizziness or vertigo in the USA in 2011, 25.7% were attributable to otological/vestibular causes, costing US $757 million.

Although the immediate and most obvious effects of poor vestibular function are oscillopsia and ataxia (see Curthoys and Halmagyi, 1995 for a review), vestibular dysfunction results in a complex neurological syndrome characterized not only by reflex deficits, but also by spatial memory deficits, autonomic and anxiety disorders (see Smith and Curthoys, 1989; Smith et al., 2010 and Gurvich et al., 2013, in press, for reviews). In one of the first clinical studies, Grimm et al. (1989) described patients with a perilymph fistular syndrome (a rupture in the labyrinth, resulting in leakage of perilymphatic fluid) who experienced not only vestibular symptoms (e.g., positional vertigo) but also a variety of cognitive and emotional symptoms, including memory and attention deficits, anxiety and depression. Since then, many papers have been published reporting spatial memory and attention deficits in patients with different kinds of vestibular disorders (Risey and Briner, 1990–1991; Peruch et al., 1999; Yardley et al., 2001, 2002; see Gizzi et al., 2003 for evidence to the contrary; Schautzer et al., 2003; Black et al., 2004; Borel et al., 2004; Redfern et al., 2004; Brandt et al., 2005; Talkowski et al., 2005; see Smith et al., 2005b; Hanes and McCollum, 2006; Guidetti et al., 2008 for reviews). Although it is possible that cognitive dysfunction is an indirect consequence of symptoms such as vertigo, studies of patients with chronic vestibular loss, but without vertigo, have still demonstrated spatial memory impairment (Guidetti et al., 2008). In addition, a large body of evidence from animal studies has demonstrated that animals with vestibular lesions suffer from cognitive impairment (Potegal et al., 1977; Horn et al., 1981; Potegal, 1982; Miller et al., 1983; Petrosini, 1984; Mathews et al., 1988, 1989; Semenov and Bures, 1989; Schaeppi et al., 1991; Chapuis et al., 1992; Ossenkopp and Hargreaves, 1993; Stackman and Herbert, 2002; Wallace et al., 2002; Russell et al., 2003a; Zheng et al., 2003, 2004, 2006, 2007, 2008, 2009a,b, 2012; Baek et al., 2010; Besnard et al., 2012; Machado et al., 2012; Smith et al., 2013).

It is clear that the loss of vestibular information results in the abnormal function of many brain regions, including the hippocampus, which has been demonstrated to atrophy following complete bilateral vestibular loss in humans (Brandt et al., 2005). However, exactly why this happens is unclear. The vestibular system encodes angular and linear acceleration of the head in three dimensions and, in addition to generating the VORs and VSRs, provides the brain with information about self-motion that can be used to navigate through the environment and form memories for places in it (Smith et al., 2010; Cullen, 2012). However, the severity of the symptoms of vestibular dysfunction suggests that there may be a critical dependence of the brain and body upon vestibular input. The most primitive part of the vestibular system—the otoliths that transduce linear acceleration, including linear acceleration by gravity—is estimated to be more than 500 million years old and exists in primitive species such as sea squirts (Smith et al., 2010). These sensory organs evolved to provide information about gravitational vertical, before any other sensory system had developed, and during development the vestibular system is fully functional before the visual or auditory systems. Therefore, it is highly likely that human physiology has developed a special dependence upon the otolithic part of the vestibular system (the utricle and saccule in mammals; Smith et al., 2010).

Vestibular dysfunction in humans is often associated with anxiety disorders, including panic attacks and phobias, as well as depression (Eagger et al., 1992; Asmundson et al., 1998; Balaban and Thayer, 2001; Furman and Jacob, 2001; Monzani et al., 2001; Balaban, 2002; Grunfeld et al., 2003; Persoons et al., 2003; Pollak et al., 2003; Godemann et al., 2004a,b; Best et al., 2006; Staab, 2006; Godemann et al., 2009; Gurvich et al., 2013, in press). While it is possible that anxiety is a direct consequence of vestibular dysfunction, it has also been reported that anxiety disorders can cause dizziness of vestibular origin (Asmundson et al., 1998; Venault et al., 2001; Bolmont et al., 2002; Staab et al., 2002; Tecer et al., 2004; Best et al., 2006; Furman et al., 2006) and antidepressants such as selective serotonin reuptake inhibitors (SSRIs) have been reported to relieve dizziness associated with psychiatric symptoms (Staab et al., 2002; Simon et al., 2005; Horii et al., 2007). It is possible that emotional disorders arise indirectly from cognitive impairment. However, Halberstadt and Balaban (2006) have reported that the same neurons in the dorsal raphe nucleus that release serotonin, send projections into the amygdala, an area of the brain concerned with fear and panic, as well as the brainstem vestibular nucleus (VN). This finding suggests that changes in emotional tone may directly influence the vestibular system. This kind of evidence indicates that vestibular impairment may cause a multitude of changes in cognition, emotion and personality, which is consistent with some evidence that vestibular disease is associated with unusually high rates of depersonalization/derealization symptoms, which include “difficulty focussing attention” and “thoughts seeming blurred” (Sang et al., 2006; Jauregui-Renaud et al., 2008a,b).

The aim of this review is to summarize and evaluate evidence relating to the contribution of the vestibular system to aspects of personality that are beyond the cognitive and emotional effects of vestibular loss that have already been well documented. For this reason we will not review the studies of anxiety and depression associated with vestibular dysfunction that have been reviewed elsewhere (e.g., Asmundson et al., 1998; Balaban and Thayer, 2001; Furman and Jacob, 2001; Balaban, 2002; Staab, 2006; Gurvich et al., 2013, in press) but focus instead on other aspects of personality such as depersonalization and derealization symptoms, which were reported in patients with Meniere’s disease, as early as 1989 (Grigsby and Johnston, 1989). We believe that these studies provide additional insights into the role that vestibular sensation plays in the conceptualization of the self.

## Personality and vestibular function

**Figure 1:**
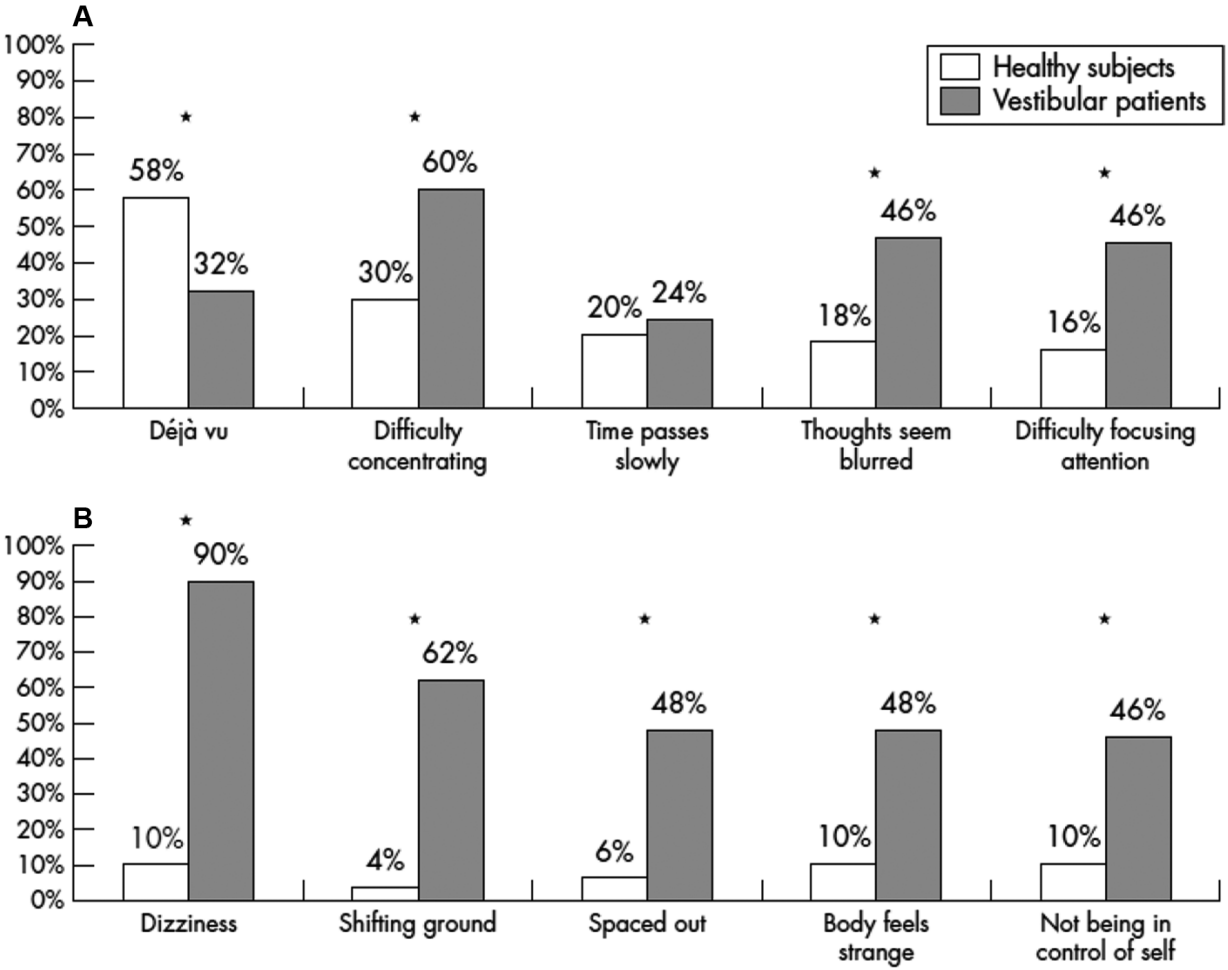
**Percentage of vestibular patients and healthy controls reporting the five most frequent depersonalization and derealization symptoms**. Reproduced from Sang et al. (2006) with permission.

 A number of interesting studies have been published on depersonalization and derealization symptoms in patients with vestibular disorders. Sang et al. (2006) studied 50 patients with peripheral vestibular disorders and compared them to 121 healthy subjects using the 28 item depersonalization and derealization inventory of Cox and Swinson (2002). Both the frequency and the severity of depersonalization and derealization symptoms was significantly greater in patients with vestibular disorders than in the controls, including experiences such as “feeling as if walking on shifting ground”, “body feels strange/not being in control of self” and “feeling spacey” or “spaced out”. Caloric stimulation of healthy subjects was found to induce depersonalization and derealization symptoms that they did not otherwise experience, whereas in the patients the induced symptoms were similar to those that they had already experienced. Figures 1A,B shows the significant increase in the incidence of symptoms such as “difficulty concentrating”, “thought seeming blurred”, “difficulty focussing attention”, “shifting ground”, “spaced out”, “body feels strange” and “not being in control of self” in vestibular patients compared to healthy controls. Similar results were published by Jauregui-Renaud et al. (2008a,b). Jauregui-Renaud et al. (2008a) reported that the most severe depersonalization and derealization scores were observed in patients with bilateral vestibular loss and the least severe scores were in patients with unilateral canal paresis without balance symptoms. The severity of the symptoms was significantly related to the degree of estimation error in a spatial orientation task (*P* ≤ 0.05), although the *R*^2^ was low (0.25). In a further study, the authors found that the number and severity of depersonalization and derealization symptoms were greater in patients with retinal disease or vestibular dysfunction, compared to those with hearing disorders or in healthy controls (Jauregui-Renaud et al., 2008b).

Recently, Gómez-Alvarez and Jáuregui-Renaud (2011) have reported the results of a study of 10 patients whom they studied during the first 3 months following a unilateral vestibular lesion. The patients exhibited depersonalization and derealization symptoms that were statistically related to their impairment of balance.

 Unlike the spatial memory deficits that have been documented in humans with bilateral vestibular loss (e.g., Brandt et al., 2005), it is difficult if not impossible to dissociate the depersonalization and derealization symptoms such as feeling “spaced out”, “body feels strange” and “not being in control of self”, from symptoms that are a direct consequence of the impairment of the VORs and VSRs, such as oscillopsia and ataxia. Indeed, these patients often complain of dizziness (Sang et al., 2006; see Figure 1B).

Nonetheless, the presence of depersonalization and derealization symptoms in patients with vestibular dysfunction suggests that vestibular sensation contributes to the definition of the self, in terms of the sense of where the body is in relation to the external world. The vestibular system is one of a number of sensory systems that contributes to the internal representation of the self and can promote feelings of disembodiment when dysfunction occurs (see Giummarra et al., 2008 and Lopez et al., 2008 for reviews; Cheyne and Girard, 2009; Terhune, 2009; Pfeiffer et al., 2013). Lopez and colleagues have reported that vestibular stimulation can modify the body schema and even the sense of ownership of an illusory hand in the “rubber hand illusion” (Lopez et al., 2010, 2012).

In one of the most unusual studies to date, Lopez et al. (2013) investigated whether observing someone else in motion could influence a person’s perception of self-motion. Subjects were rotated in a leftward or rightward direction while viewing videos of their own body, another body or an object rotating in the yaw plane. They found that the response time to correctly identify the subject’s direction of self-motion was significantly increased for videos involving incongruent self-motion and incongruent object motion, and this effect was reduced for observation of someone else’s motion. Interestingly, the “congruency effect” was correlated with subjects’ empathy scores (*P* ≤ 0.05), although the correlation coefficients were quite low (*r* = 0.48). The authors concluded that the results suggest the existence of a “vestibular mirror neuron system”.

## Neuronal substrates of the vestibular contribution to self

Stackman et al. (2002) reported that reversible, bilateral inactivation of the vestibular labyrinth, using intratympanic tetrodotoxin, resulted in a loss of the selective firing of hippocampal neurons that encode places in the environment (“place cells”) in alert rats, a result replicated by Russell et al. (2003b) using permanent bilateral surgical lesions of the labyrinth. Russell et al. (2006) also reported that bilateral labyrinthectomy caused a disruption of theta rhythm, which is believed to coordinate the activity of hippocampal place cells (see also Neo et al., 2012; Tai et al., 2012). Taken together, these data from animal studies support the view that vestibular information is fundamentally important for the generation of hippocampal spatial memories in animals and humans (Smith and Curthoys, 1989; Smith et al., 2005a, 2010). There is still debate as to how vestibular information reaches the hippocampus. The thalamus is certain to be one important relay station for at least some vestibular information; however, the number of different vestibulo-hippocampal pathways and their precise nature, remains to be determined (Smith, 1997; see Shinder and Taube, 2010 for a recent review). However, the hippocampus is only one part of a highly complex system of limbic-neocortical pathways that are responsible for spatial memory (see Guldin and Grusser, 1998; Hanes and McCollum, 2006; Shinder and Taube, 2010, for reviews). In humans, functional magnetic resonance imaging (fMRI) has revealed that areas of significant activation by galvanic vestibular stimulation (GVS) include the posterior insula, the retroinsular regions, the superior temporal gyrus, parts of the inferior parietal lobule, the intraparietal sulcus, the post-central and pre-central gyrus, the anterior insular, the inferior frontal gyrus, the anterior cingulate gyrus, the precuneus and the hippocampus (Lobel et al., 1998; see Karnath and Dieterich, 2006 for a review). Activation of cortical networks during GVS is not symmetrical; it is stronger in the non-dominant hemisphere, in the hemisphere ipsilateral to stimulated ear, and in the hemisphere ipsilateral to the fast phase of vestibular nystagmus (see Karnath and Dieterich, 2006 for a review).

While vestibular input to areas such as the hippocampus is likely to be important for the internal representation of self, neocortical regions that integrate this information with a variety of other sensory and emotional information may be critical locations. Ionta et al. (2011) used fMRI to study brain activity during a metal ball dropping task that required subjects to estimate how long it would take the ball to hit the ground. Changes in the subject’s estimate of self-location were induced by changing the synchrony between the stroking of the subject’s back and the back of a visually-presented virtual human body. They found that during changes in the perception of self-location, activity changed in the left and right temporo-parietal junction (TPJ). To further investigate this connection, they analyzed the lesion sites in neurological patients who had out-of-body experiences (OBEs). In eight out of nine OBE patients, the lesions were situated in the right temporal or parietal cortex, usually at the TPJ. It is possible, therefore, that this is a critical site for the generation of the internal representation of self-location (see Lenggenhager et al., 2006 for a review).

**Figure 2:**
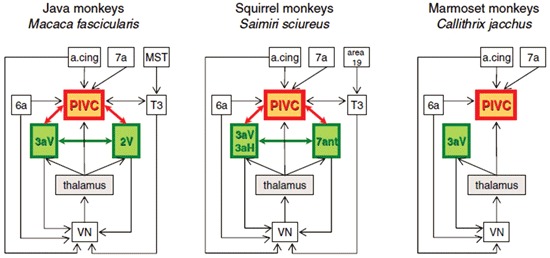
**Schematic diagram of interactions between the VN, the thalamus, and cortical regions in Old World monkeys (Java monkeys,** *Macaca fascicularis***) and New World monkeys (squirrel monkeys,** *Saimiri sciureus* **and marmoset monkeys,** *Callithrix jacchus***)**. A cing: anterior cingulate region. PIVC: parieto-insular vestibular cortex. MST: medial superior temporal area. Reproduced from Lopez and Blanke (2011) with permission.

In fact, GVS has been reported to activate the left TPJ in humans, which may be analogous to the parieto-insular vestibular cortex (PIVC) in monkeys (Lobel et al., 1998), although this is controversial (see Lopez and Blanke, 2011 for a review). Although the group analysis in Lobel et al. (1998) showed a clear lateralization of the activation to the left hemisphere, four individual subjects also showed activation in the right hemisphere. Bottini et al. (1994) also showed that cold caloric vestibular stimulation activated the posterior insular region and the posterior lateral sulcus of the TPJ. Miyamoto et al. (2007) also reported that high intensity click stimulation of the saccule activated the TPJ. Many neurons in the monkey PIVC respond to vestibular input as well as proprioceptive input from the neck (see Shinder and Taube, 2010 and Lopez and Blanke, 2011 for reviews; see Figure 2), and this information is transmitted to the ventral intraparietal cortex (VIP), a major site of multisensory integration (e.g., Chen et al., 2011).

## Conclusion

Over the last 20 years, evidence from human and animal studies has continually extended the appreciation of the contribution of the vestibular system to human consciousness. While the vestibular system was once recognized mainly for its regulation of eye movement and postural reflexes, increasingly it has become apparent that the cortical representation of vestibular information is important for cognition, emotion and even the sense of self. While it might seem self-evident that knowing where you are using vestibular information, also defines the boundary between the self and the external world, many of the more ethereal effects of vestibular dysfunction, which are difficult to describe, have gone undocumented or at least poorly recognized in the neurological literature. The publication of studies demonstrating depersonalization and derealization symptoms in patients with vestibular disorders has illuminated the fact that the vestibular system contributes to many aspects of higher consciousness and the very definition of the self (Sang et al., 2006; Jauregui-Renaud et al., 2008a,b). Recent studies suggest that the TPJ in humans and areas such as the PIVC and VIP in monkeys, may be sites of multisensory integration of vestibular information with other sensory inputs, that contribute to the generation of depersonalization and derealization symptoms. Increasingly, neurobiological studies will delineate exactly where and how this contribution takes place (Ionta et al., 2011).

## Conflict of interest statement

The authors declare that the research was conducted in the absence of any commercial or financial relationships that could be construed as a potential conflict of interest.
